# Educational games for brain health: revealing their unexplored potential through a neurocognitive approach

**DOI:** 10.3389/fpsyg.2015.01056

**Published:** 2015-07-24

**Authors:** Patrick Fissler, Iris-Tatjana Kolassa, Claudia Schrader

**Affiliations:** ^1^Institute of Psychology and Education, Clinical and Biological Psychology, Ulm University, Ulm, Germany; ^2^Institute of Psychology and Education, Serious Games, Ulm University, Ulm, Germany

**Keywords:** educational games, serious games, brain health, gaming, education, cognitive ability, brain function, brain structure

## Abstract

Educational games link the motivational nature of games with learning of knowledge and skills. Here, we go beyond effects on these learning outcomes. We review two lines of evidence which indicate the currently unexplored potential of educational games to promote brain health: First, gaming with specific neurocognitive demands (e.g., executive control), and second, educational learning experiences (e.g., studying foreign languages) improve brain health markers. These markers include cognitive ability, brain function, and brain structure. As educational games allow the combination of specific neurocognitive demands with educational learning experiences, they seem to be optimally suited for promoting brain health. We propose a neurocognitive approach to reveal this unexplored potential of educational games in future research.

## The Power of Educational Games

Playing games is one of the most popular leisure activities. For example, 59% of Americans play video games (Entertainment Software Association, 2014). In contrast to watching a video or reading a book, video games afford interactive exploration and challenge due to user control, competition, levels of difficulty, and reward (Malone, 1981; Lucas and Sherry, 2004). These design characteristics are essential for player’s motivation in games (Sweetser and Wyeth, 2005).

*Educational games* aim to use this motivational quality of games for educationally relevant learning purposes (knowledge and skill acquisition). They are a branch of *serious games* which are defined as “games that do not have entertainment, enjoyment or fun as their primary purpose” (Michael and Chen, 2006, p. 21). Domains of learning include history, engineering, biology, math, and language (Young et al., 2012; Wouters et al., 2013). For example, *Re-mission* aims to improve cancer-related knowledge (Beale et al., 2007) and *Twelve a Dozen*^1^ teaches mathematical operations. The number of these games increased exponentially since the 1990s in industry and in research (Laamarti et al., 2014). A recent meta-analysis by Wouters et al. (2013) investigated the effectiveness of educational games in terms of learning. It included 77 studies with more than 5,500 participants and found that the games induced even more knowledge and skill acquisition than conventional instruction methods.

In this perspective article, however, we go beyond educational games’ effects on learning of knowledge and skills (i.e., plasticity of representations, cf. Craik and Bialystok, 2006; Lövdén et al., 2010). We review research which suggests the currently unexplored potential of educational games for brain health (i.e., plasticity of processes, cf. Craik and Bialystok, 2006; Lövdén et al., 2010) and propose a neurocognitive approach to reveal this potential.

**FIGURE 1:**
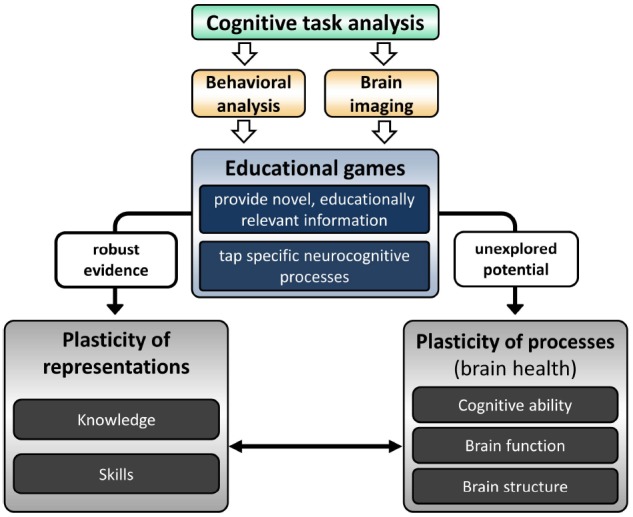
**A neurocognitive approach to reveal the unexplored potential of educational games for brain health.** In a two-step approach, a cognitive task analysis of educational games is followed by their validation through objective methods. This second step consists of a behavioral analysis to determine the association between game performance and neuropsychological test performance and/or a brain imaging approach to determine the recruited neuronal networks for task completion. Based on this approach, appropriate educational games can be selected to enable randomized controlled clinical trials that assess the efficacy of educational games to improve brain health markers including cognitive ability, brain function, and brain structure.

First, we briefly review evidence for the beneficial effect of games with specific neurocognitive demands on brain health. Second, we depict the positive impact of educationally relevant learning experiences on brain health. Educational games enable both specific neurocognitive demands and educational learning experience. However, to our knowledge, there are currently no studies exploring their potential for brain health. Hence, in the last section, we propose a two-step neurocognitive approach to identify appropriate educational games that should be rigorously tested in randomized controlled clinical trials (see Figure 1).

## Gaming with Specific Neurocognitive Demands Promotes Brain Health

The supply-demand mismatch model of cognitive plasticity assumes that neurocognitive demands which are greater than the brain’s functional supply induce beneficial neuroplastic changes (Lövdén et al., 2010). It is assumed that this supply-demand mismatch needs to be prolonged (at least more than several hours for small effect sizes) to overcome the inertia and sluggishness of plasticity (Lövdén et al., 2010). Games can pose prolonged neurocognitive demands on working memory, perceptual speed, and episodic memory (Baniqued et al., 2013). Thus, games might induce respective neurocognitive benefits. For example, games that heavily tap executive control processes such as working memory are thought to induce positive plastic changes in these cognitive processes and their underlying prefrontal network. Such changes may range from more efficient brain function (Bavelier et al., 2012; Anguera et al., 2013) to benefits in brain structure such as increases in gray matter volume (Kühn et al., 2013), cortical thickness (Kühn et al., 2014), and neurotransmitter receptors (McNab et al., 2009).

Current advances in gaming research support the supply-demand mismatch model (see Powers et al., 2013; Bisoglio et al., 2014 for a meta-analysis and a review). Cognitively demanding digital games as well as non-digital board and card games improved cognitive abilities (Cheng et al., 2013; Fissler et al., 2013; Powers et al., 2013). These gaming-induced benefits comprised lower-order abilities such as visual perception (Green and Bavelier, 2012) and higher-order abilities such as selective visual attention (Green and Bavelier, 2003), switching ability (Basak et al., 2008; Green et al., 2012; Strobach et al., 2012), sustained attention (Anguera et al., 2013), short-term and working memory (Basak et al., 2008; Anguera et al., 2013; Cheng et al., 2013), executive control (Fissler et al., 2013), reasoning, and spatial abilities (Feng et al., 2007; Shute et al., 2015).

For example, a study by Shute et al. (2015) showed that a commercial off-the-shelf game called *Portal 2* with process-specific demands on spatial reasoning improved cognitive abilities even more than an intentionally-designed cognitive training program (i.e., repeated practice of standardized cognitive task paradigms for specific cognitive abilities with adapting difficulty levels, Gates and Valenzuela, 2010). In contrast to participants following the cognitive training program, *Portal 2* players improved more in performance on non-trained problem solving and spatial ability tests. Furthermore, playing the video game was more enjoyable than the cognitive training program (Shute et al., 2015).

Recent studies provide first insights into the neuronal underpinning of game-induced cognitive benefits. They range from plastic changes in brain structure to brain function. Kühn et al. (2013) found that playing *Super Mario 64* increased gray matter of the right hippocampal formation and dorsolateral prefrontal cortex as well as of the cerebellum bilaterally. These brain areas are known to play an essential role in spatial memory, executive function, and fine-tuned motor skills. Using electrophysiological methods, Anguera et al. (2013) demonstrated functional brain benefits in the prefrontal cognitive control system through a dual-task driving game called *NeuroRacer*. Importantly, non-trained neuropsychological test performance improved through training and these gains were positively associated with the neurofunctional changes.

These experimental findings are backed by observational studies on the association of gaming with brain health markers. Frequent players of board games, in contrast to rare players, showed a reduced cognitive decline and incidence of dementia (Verghese et al., 2003; Dartigues et al., 2013). Bavelier et al. (2012) investigated associations of brain function with gaming experience. Frequent gamers, in contrast to non-gamers, showed reduced neuronal recruitment of the fronto-parietal network in attentionally challenging tasks which indicates more efficient attentional processing. Finally, associations of gaming with brain structure were recently revealed. The duration of video gaming (hours per week) was positively associated with left prefrontal cortical thickness (Kühn et al., 2014). The number of years spent video gaming was positively related to entorhinal cortex, hippocampal, and occipital gray matter volume (Kühn and Gallinat, 2014).

In sum, these recent advances in gaming research emphasize the potential of cognitively challenging games to improve different markers of brain health ranging from cognitive ability, brain function, and brain structure to incidence of dementia. In the following, we will outline the impact of educationally relevant learning of knowledge and skills on brain health markers.

## Educational Learning Experiences Promote Brain Health

Extensive learning experiences are thought to require prolonged activation of basic neurocognitive abilities such as executive control processes and long-term memory (Park et al., 2014). These prolonged neurocognitive demands may induce positive plastic changes in accordance with the supply-demand mismatch model (Lövdén et al., 2010). Successful learning experiences may enhance brain health by additional mechanisms as evidenced in animal models. Learning novel information increased survival of newborn cells in the hippocampus, an area that plays an essential role for episodic memory (see Shors, 2014, for a review). In addition, intrinsic plasticity—a metaplasticity mechanism which changes the threshold for intrinsic neuronal excitability—is increased in the hippocampus through successful learning experiences (see Sehgal et al., 2013, for a review). Furthermore, an enriched environment, which provides a range of learning opportunities, reduced pathological processes that are associated with Alzheimer’s disease (Lazarov et al., 2005; Costa et al., 2007) and reduced the detrimental effect of Alzheimer’s disease-related Aβ oligomers on long-term potentiation (Li et al., 2013).

A positive effect of educationally relevant learning experiences on markers of brain health has also been found in experimental studies with humans. Diverse interventions targeting at knowledge and skill acquisition improved cognitive abilities. Extensive learning experiences within a senior computer course improved working memory and episodic memory (Klusmann et al., 2010). A digital-photography and quilting course improved episodic memory (Park et al., 2014). A tablet course improved speed and episodic memory (Chan et al., 2014) and extensive training of a foreign language enhanced associative memory (Mårtensson and Lövdén, 2011). For example, Park et al. (2014) investigated the cognitive benefits of acquiring digital-photography skills by the use of a single-lens reflex camera and photo-editing software 15 h a week for 3 months. Compared to a group which completed activities that relied on activation of prior knowledge (e.g., listening to music, watching DVDs, or completing word-meaning puzzles), their episodic memory performance improved more (Park et al., 2014).

In addition, extensive educational learning interventions induced plasticity in brain function (i.e., increased activity in the anterior cingulum, Carlson et al., 2009) and brain structure (Draganski et al., 2006; Woollett and Maguire, 2011). The hippocampus increased in volume after extensive learning for medical exams (Draganski et al., 2006) and after successful training for a London taxi driver license (Woollett and Maguire, 2011).

This interventional evidence is backed by robust observational evidence regarding the relationship of education with brain health markers. Strong positive associations between years spent in education and risk for cognitive decline (Valenzuela and Sachdev, 2006) as well as dementia (Caamaño-Isorna et al., 2006) have been demonstrated. Low education represents the single most preventable risk factor for Alzheimer’s dementia. Worldwide, 19% of affected individuals are potentially attributable to low education (Barnes and Yaffe, 2011). In addition, acquisition of skills such as speaking a second language and playing a musical instrument predicted a reduced future risk of cognitive decline (Bak et al., 2014) and dementia (see e.g., Balbag et al., 2014, for a population-based twin study). Furthermore, more years spent in education was associated with greater brain weight (Brayne et al., 2010) and, in one pilot-study, also with lower markers of Alzheimer’s disease pathology (Yasuno et al., 2014).

In the last two sections, we reviewed evidence for beneficial effects on brain health a) through gaming-induced neurocognitive demands and b) through educationally relevant learning of knowledge and skills. As educational games allow the combination of both, they seem to be optimally suited to promote brain health. However, to our knowledge, no study investigated the impact of educational gaming on brain health markers, yet. Hence, we propose a two-step neurocognitive approach in the following section that aims to reveal their unexplored potential.

## A Neurocognitive Approach to Reveal the Potential of Educational Games for Brain Health

We have outlined above that games which induce learning of novel information and pose specific neurocognitive demands seem to be optimally-suited for brain health purposes. Clearly, not all educational games pose specific neurocognitive demands and appropriate games need to be identified from the large and growing market (cf. Wartella, 2015). We propose a two-step approach to elucidate the neurocognitive demands of educational games (see Figure 1).

In the first step, a cognitive task analysis should be conducted for a wide range of educational games in order to determine the most appropriate cognitively challenging games for the more cost-intensive second step. Cognitive task analysis is a set of methods aiming to determine the cognitive demands to perform a task proficiently (Militello and Hutton, 1998). We briefly depict one approach of a cognitive task analysis suited for educational games (cf. Baniqued et al., 2013) and exemplify this method with *DragonBox2*, an educational game which aims to teach algebra in a fun way^2^.

First, a game diagram is created to determine the cognitively demanding tasks of the respective educational game. Here, an expert for the game (1) breaks the game down into its major tasks (usually between one and five tasks) and (2) determines which tasks pose substantial demands on cognitive abilities such as attention, speed or memory (cf. task diagram method; Militello and Hutton, 1998). In *DragonBox2*, there is one major task (i.e., isolating a dragon captured in a box on one side of the screen, or in other words, solving an algebraic equation for the *x*) and this task poses substantial cognitive demands.

Subsequently, neuropsychologists should rate the major tasks of appropriate educational games on their specific neurocognitive demands. The rating should be based on a validated taxonomy of neurocognitive abilities. For example, executive functions can be subdivided in three components including updating, inhibition, and shifting (Miyake et al., 2000; Miyake and Friedman, 2012). Memory can be subdivided in the two components declarative memory and procedural memory (Squire, 1992; Robertson, 2009). *DragonBox2* poses high demands on executive function (frontal brain systems) and memory (mediotemporal and basal ganglia systems). For example, monitoring multiple items which are added and deleted from working memory through the mental application of algebra rules poses demands on updating; flexibly switching between multiple algebra rules which are cued by a given stimulus set requires shifting; selecting the application of non-dominant rules instead of more prepotent rules poses demands on inhibition; knowledge acquisition for the game’s 24 algebra rules taps declarative memory; skill acquisition regarding arithmetics, factorization, or the creation of parameters poses demands on procedural memory.

In the more cost-intensive second step, two objective methods—a behavioral and/or a brain imaging approach—can be used to substantiate the assumed neurocognitive demands revealed by the cognitive task analysis. In the behavioral approach, associations between game performance and performance in neuropsychological tests are assessed (cf Jaeggi et al., 2010; Baniqued et al., 2013; Rode et al., 2014). The pattern of the game-test associations enables the validation of the games’ neurocognitive demands.

The brain imaging approach aims to reveal the neuronal networks recruited by the games. Different brain imaging methods such as electroencephalography (Anguera et al., 2013), near-infrared spectroscopy (Ekkekakis, 2009), or functional magnetic resonance imaging (Dahlin et al., 2008; Voss et al., 2012) can be used. Finally, after successful identification of appropriate educational games through behavioral analysis and/or brain imaging, long-term randomized controlled clinical trials should examine their effects on brain health markers (see Moher et al., 2010, for methodological issues).

## Conclusion

In this perspective article, we reviewed two lines of research that indicate an unexplored potential of educational games to improve brain health. First, games with specific neurocognitive demands and second, educationally relevant learning experiences positively impact brain health markers including cognitive abilities, brain function, and brain structure. Future research should use a neurocognitive approach to identify cognitively challenging educational games. These should be rigorously examined in randomized controlled long-term clinical trials regarding their effects on brain health.

### Conflict of Interest Statement

The authors declare that the research was conducted in the absence of any commercial or financial relationships that could be construed as a potential conflict of interest.

## Footnotes

1 http://www.bossastudios.com/games/twelve/

2 http://www.dragonboxapp.com/

## References

[B1] Anguera J. A. Boccanfuso J. Rintoul J. L. Al-Hashimi O. Faraji F. Janowich J. (2013). Video game training enhances cognitive control in older adults. Nature 501, 97–101. 10.1038/nature12486 24005416 PMC3983066

[B2] Bak T. H. Nissan J. J. Allerhand M. M. Deary I. J. (2014). Does bilingualism influence cognitive aging? Ann. Neurol. 75, 959–963. 10.1002/ana.24158 24890334 PMC4320748

[B3] Balbag M. A. Pedersen N. L. Gatz M. (2014). Playing a musical instrument as a protective factor against dementia and cognitive impairment: a population-based twin study. Int. J. Alzheimers Dis. 2014, 6. 10.1155/2014/836748 25544932 PMC4269311

[B4] Baniqued P. L. Lee H. Voss M. W. Basak C. Cosman J. D. Desouza S. (2013). Selling points: what cognitive abilities are tapped by casual video games? Acta Psychol. 142, 74–86. 10.1016/j.actpsy.2012.11.009 23246789 PMC3679476

[B5] Barnes D. E. Yaffe K. (2011). The projected effect of risk factor reduction on Alzheimer’s disease prevalence. Lancet Neurol. 10, 819–828. 10.1016/S1474-4422(11)70072-2 21775213 PMC3647614

[B6] Basak C. Boot W. R. Voss M. W. Kramer A. F. (2008). Can training in a real-time strategy video game attenuate cognitive decline in older adults? Psychol. Aging 23, 765–777. 10.1037/a0013494 19140648 PMC4041116

[B7] Bavelier D. Achtman R. L. Mani M. Föcker J. (2012). Neural bases of selective attention in action video game players. Vision Res. 61, 132–143. 10.1016/j.visres.2011.08.007 21864560 PMC3260403

[B8] Beale I. L. Kato P. M. Marin-Bowling V. M. Guthrie N. Cole S. W. (2007). Improvement in cancer-related knowledge following use of a psychoeducational video game for adolescents and young adults with cancer. J. Adolesc. Health 41, 263–270. 10.1016/j.jadohealth.2007.04.006 17707296

[B9] Bisoglio J. Michaels T. I. Mervis J. E. Ashinoff B. K. (2014). Cognitive enhancement through action video game training: great expectations require greater evidence. Front. Psychol. 5:136. 10.3389/fpsyg.2014.00136 24600427 PMC3928536

[B10] Brayne C. Ince P. G. Keage H. a. D. Mckeith I. G. Matthews F. E. Polvikoski T. (2010). Education, the brain and dementia: neuroprotection or compensation? Brain 133, 2210–2216. 10.1093/brain/awq185 20826429

[B11] Caamaño-Isorna F. Corral M. Montes-Martínez A. Takkouche B. (2006). Education and dementia: a meta-analytic study. Neuroepidemiology 26, 226–232. 10.1159/000093378 16707907

[B12] Carlson M. Erickson K. Kramer A. Voss M. Bolea N. Mielke M. (2009). Evidence for neurocognitive plasticity in at-risk older adults: the experience corps program. J. Gerontol. Series A Biol. Sci. Med. Sci. 64, 1275–1282. 10.1093/gerona/glp117 19692672 PMC2781785

[B13] Chan M. Y. Haber S. Drew L. M. Park D. C. (2014). Training older adults to use tablet computers: does it enhance cognitive function? Gerontologist [Epub ahead of print]. 10.1093/geront/gnu057 24928557 PMC4873760

[B14] Cheng S.-T. Chow P. K. Song Y.-Q. Yu E. Chan A. Lee T. (2013). Mental and physical activities delay cognitive decline in older persons with dementia. Am. J. Geriatr. Psychiatry 10, 1–13. 10.1016/j.jagp.2013.01.060 23582750

[B15] Costa D. A. Cracchiolo J. R. Bachstetter A. D. Hughes T. F. Bales K. R. Paul S. M. (2007). Enrichment improves cognition in AD mice by amyloid-related and unrelated mechanisms. Neurobiol. Aging 28, 831–844. 10.1016/j.neurobiolaging.2006.04.009 16730391

[B16] Craik F. Bialystok E. (2006). Cognition through the lifespan: mechanisms of change. Trends Cogn. Sci. 10, 131–138. 10.1016/j.tics.2006.01.007 16460992

[B17] Dahlin E. Neely A. S. Larsson A. Backman L. Nyberg L. (2008). Transfer of learning after updating training mediated by the striatum. Science 320, 1510–1512. 10.1126/science.1155466 18556560

[B18] Dartigues J. F. Foubert-Samier A. Le Goff M. Viltard M. Amieva H. Orgogozo J. M. (2013). Playing board games, cognitive decline and dementia: a French population-based cohort study. BMJ Open 3, e002998. 10.1136/bmjopen-2013-002998 23988362 PMC3758967

[B19] Draganski B. Gaser C. Kempermann G. Kuhn H. G. Winkler J. Buchel C. (2006). Temporal and spatial dynamics of brain structure changes during extensive learning. J. Neurosci. 26, 6314–6317. 10.1523/JNEUROSCI.4628-05.2006 16763039 PMC6675198

[B20] Ekkekakis P. (2009). Illuminating the black box: investigating prefrontal cortical hemodynamics during exercise with near-infrared spectroscopy. J. Sport Exerc. Psychol. 31, 505–553. 19842545 10.1123/jsep.31.4.505

[B21] Entertainment Software Association. (2014). Essential Facts About the Computer and Video Game Industry. Available at: http://www.theesa.com/wp-content/uploads/2014/10/ESA_EF_2014.pdf

[B22] Feng J. Spence I. Pratt J. (2007). Playing an action video game reduces gender differences in spatial cognition. Psychol. Sci. 18, 850–855. 10.1111/j.1467-9280.2007.01990.x 17894600

[B23] Fissler P. Küster O. Schlee W. Kolassa I. T. (2013). “Novelty interventions to enhance broad cognitive abilities and prevent dementia: synergistic approaches for the facilitation of positive plastic change,” in Progress in Brain Research, eds Merzenich M. M. Nahum M. Van Vleet T. M. (Oxford: Elsevier), 403–434. 10.1016/B978-0-444-63327-9.00017-5 24309264

[B24] Gates N. J. Valenzuela M. (2010). Cognitive exercise and its role in cognitive function in older adults. Curr. Psychiatry Rep. 12, 20–27. 10.1007/s11920-009-0085-y 20425306

[B25] Green C. Bavelier D. (2012). Learning, attentional control, and action video games. Curr. Biol. 22, R197–R206. 10.1016/j.cub.2012.02.012 22440805 PMC3461277

[B26] Green C. S. Bavelier D. (2003). Action video game modifies visual selective attention. Nature 423, 534–537. 10.1038/nature01647 12774121

[B27] Green C. S. Sugarman M. A. Medford K. Klobusicky E. Bavelier D. (2012). The effect of action video game experience on task-switching. Comput. Human Behav. 28, 984–994. 10.1016/j.chb.2011.12.020 22393270 PMC3292256

[B28] Jaeggi S. M. Studer-Luethi B. Buschkuehl M. Su Y. F. Jonides J. Perrig W. J. (2010). The relationship between n-back performance and matrix reasoning—implications for training and transfer. Intelligence 38, 625–635. 10.1016/j.intell.2010.09.001

[B29] Klusmann V. Evers A. Schwarzer R. Schlattmann P. Reischies F. Heuser I. (2010). Complex mental and physical activity in older women and cognitive performance: a 6-month randomized controlled trial. J. Gerontol. A Biol. Sci. Med. Sci. 65, 680–688. 10.1093/gerona/glq053 20418350

[B30] Kühn S. Gallinat J. (2014). Amount of lifetime video gaming is positively associated with entorhinal, hippocampal and occipital volume. Mol. Psychiatry 19, 842–847. 10.1038/mp.2013.100 23958958

[B31] Kühn S. Gleich T. Lorenz R. C. Lindenberger U. Gallinat J. (2013). Playing Super Mario induces structural brain plasticity: gray matter changes resulting from training with a commercial video game. Mol. Psychiatry 19, 265–271. 10.1038/mp.2013.120 24166407

[B32] Kühn S. Lorenz R. Banaschewski T. Barker G. J. Büchel C. Conrod P. J. (2014). Positive association of video game playing with left frontal cortical thickness in adolescents. PLoS ONE 9:e91506. 10.1371/journal.pone.0091506 24633348 PMC3954649

[B33] Laamarti F. Eid M. El Saddik A. (2014). An overview of serious games. Int. J. Comput. Games Technol. 2014, 15 10.1155/2014/358152

[B34] Lazarov O. Robinson J. Tang Y. P. Hairston I. S. Korade-Mirnics Z. Lee V. M. Y. (2005). Environmental enrichment reduces Aβ levels and amyloid deposition in transgenic mice. Cell 120, 701–713. 10.1016/j.cell.2005.01.015 15766532

[B35] Li S. Jin M. Zhang D. Yang T. Koeglsperger T. Fu H. (2013). Environmental novelty activates β2-adrenergic signaling to prevent the impairment of hippocampal LTP by Aβ oligomers. Neuron 77, 929–941. 10.1016/j.neuron.2012.12.040 23473322 PMC3596823

[B36] Lövdén M. Bäckman L. Lindenberger U. Schaefer S. Schmiedek F. (2010). A theoretical framework for the study of adult cognitive plasticity. Psychol. Bull. 136, 659–676. 10.1037/a0020080 20565172

[B37] Lucas K. Sherry J. L. (2004). Sex differences in video game play: a communication-based explanation. Commun. Res. 31, 499–523. 10.1177/0093650204267930

[B38] Malone T. W. (1981). Toward a theory of intrinsically motivating instruction. Cogn. Sci. 5, 333–369. 10.1207/s15516709cog0504_2

[B39] Mårtensson J. Lövdén M. (2011). Do intensive studies of a foreign language improve associative memory performance? Front. Psychol. 2:12. 10.3389/fpsyg.2011.00012 21738515 PMC3125529

[B40] McNab F. Varrone A. Farde L. Jucaite A. Bystritsky P. Forssberg H. (2009). Changes in cortical dopamine D1 receptor binding associated with cognitive training. Science 323, 800–802. 10.1126/science.1166102 19197069

[B41] Michael D. R. Chen S. L. (2006). Serious Games: Games that Educate, Train, and Inform. Boston, MA: Thomson Course Technology.

[B42] Militello L. G. Hutton R. J. (1998). Applied Cognitive Task Analysis (ACTA): a practitioner’s toolkit for understanding cognitive task demands. Ergonomics 41, 1618–1641. 10.1080/001401398186108 9819578

[B43] Miyake A. Friedman N. P. (2012). The nature and organization of individual differences in executive functions: four general conclusions. Curr. Dir. Psychol. Sci. 21, 8–14. 10.1177/0963721411429458 22773897 PMC3388901

[B44] Miyake A. Friedman N. P. Emerson M. J. Witzki A. H. Howerter A. Wager T. D. (2000). The unity and diversity of executive functions and their contributions to complex. Cogn. Psychol. 41, 49–100. 10.1006/cogp.1999.0734 10945922

[B45] Moher D. Hopewell S. Schulz K. F. Montori V. Gøtzsche P. C. Devereaux P. J. (2010). CONSORT 2010 Explanation and Elaboration: updated guidelines for reporting parallel group randomised trials. BMJ 340, c869. 10.1136/bmj.c869 20332511 PMC2844943

[B46] Park D. C. Lodi-Smith J. Drew L. Haber S. Hebrank A. Bischof G. N. (2014). The impact of sustained engagement on cognitive function in older adults: the synapse project. Psychol. Sci. 25, 103–112. 10.1177/0956797613499592 24214244 PMC4154531

[B47] Powers K. Brooks P. Aldrich N. Palladino M. Alfieri L. (2013). Effects of video-game play on information processing: a meta-analytic investigation. Psychon. Bull. Rev. 20, 1055–1079. 10.3758/s13423-013-0418-z 23519430

[B48] Robertson E. M. (2009). From creation to consolidation: a novel framework for memory processing. PLoS Biol. 7:e1000019. 10.1371/journal.pbio.1000019 19175290 PMC2631067

[B49] Rode C. Robson R. Purviance A. Geary D. C. Mayr U. (2014). Is working memory training effective? A study in a school setting. PLoS ONE 9:e104796. 10.1371/journal.pone.0104796 25162637 PMC4146527

[B50] Sehgal M. Song C. Ehlers V. L. Moyer J. R. Jr. (2013). Learning to learn—Intrinsic plasticity as a metaplasticity mechanism for memory formation. Neurobiol. Learn. Mem. 105, 186–199. 10.1016/j.nlm.2013.07.008 23871744 PMC3855019

[B51] Shors T. J. (2014). The adult brain makes new neurons, and effortful learning keeps them alive. Curr. Dir. Psychol. Sci. 23, 311–318. 10.1177/0963721414540167

[B52] Shute V. J. Ventura M. Ke F. (2015). The power of play: the effects of Portal 2 and Lumosity on cognitive and noncognitive skills. Comput. Educ. 80, 58–67. 10.1016/j.compedu.2014.08.013

[B53] Squire L. R. (1992). Memory and the hippocampus: a synthesis from findings with rats, monkeys, and humans. Psychol. Rev. 99, 195–231. 10.1037/0033-295X.99.2.195 1594723

[B54] Strobach T. Frensch P. A. Schubert T. (2012). Video game practice optimizes executive control skills in dual-task and task switching situations. Acta Psychol. 140, 13–24. 10.1016/j.actpsy.2012.02.001 22426427

[B55] Sweetser P. Wyeth P. (2005). GameFlow: a model for evaluating player enjoyment in games. Comput. Entertain. 3, 3 10.1145/1077246.1077253

[B56] Valenzuela M. J. Sachdev P. (2006). Brain reserve and cognitive decline: a non-parametric systematic review. Psychol. Med. 36, 1065–1073. 10.1017/S0033291706007744 16650343

[B57] Verghese J. Lipton R. Katz M. Hall C. Derby C. Kuslansky G. (2003). Leisure activities and the risk of dementia in the elderly. N. Engl. J. Med. 348, 2508–2516. 10.1056/NEJMoa022252 12815136

[B58] Voss M. W. Prakash R. S. Erickson K. I. Boot W. R. Basak C. Neider M. B. (2012). Effects of training strategies implemented in a complex videogame on functional connectivity of attentional networks. Neuroimage 59, 138–148. 10.1016/j.neuroimage.2011.03.052 21440644

[B59] Wartella E. (2015). Educational apps: what we do and do not know. Psychol. Sci. Public Interest 16, 1–2. 10.1177/1529100615578662 25985467

[B60] Woollett K. Maguire E. A. (2011). Acquiring the knowledge of London’s layout drives structural brain changes. Curr. Biol. 21, 2109–2114. 10.1016/j.cub.2011.11.018 22169537 PMC3268356

[B61] Wouters P. Van Nimwegen C. Van Oostendorp H. Van Der Spek E. D. (2013). A meta-analysis of the cognitive and motivational effects of serious games. J. Educ. Psychol. 105, 249–265. 10.1037/a0031311

[B62] Yasuno F. Kazui H. Morita N. Kajimoto K. Ihara M. Taguchi A. (2014). Low amyloid-β deposition correlates with high education in cognitively normal older adults: a pilot study. Int. J. Geriatr. Psychiatry. 10.1002/gps.4235 [Epub ahead of print]. 25425062

[B63] Young M. F. Slota S. Cutter A. B. Jalette G. Mullin G. Lai B. (2012). Our princess is in another castle: a review of trends in serious gaming for education. Rev. Edu. Res. 82, 61–89. 10.3102/0034654312436980

